# P-455. Title: Septic Arthritis in Children: Clinical Features, Microbiologic Diagnosis, and Outcomes in a Tertiary Center Cohort in Costa Rica

**DOI:** 10.1093/ofid/ofaf695.670

**Published:** 2026-01-11

**Authors:** Monica Mendez-Mendez, Cristian Perez-Corrales, Kattia Camacho-Badilla, Maria l Avila-Aguero, Alejandra Soriano-Fallas, Mariana Vilchez-Leon, Gabriela Naranjo-Zuniga, Max Mendez-Salazar, Helena Brenes-Chacon

**Affiliations:** Hospital Nacional de Ninos "Dr. Carlos Saenz Herrera", San Jose, San Jose, Costa Rica; Hospital Nacional de Ninos "Dr. Carlos Saenz Herrera", San Jose, San Jose, Costa Rica; Caja Costarricense del Seguro Social, San Jose, San Jose, Costa Rica; Hospital Nacional de Ninos "Dr. Carlos Saenz Herrera", San Jose, San Jose, Costa Rica; Hospital Nacional de Ninos "Dr. Carlos Saenz Herrera", San Jose, San Jose, Costa Rica; Hospital Nacional de Ninos "Dr. Carlos Saenz Herrera", San Jose, San Jose, Costa Rica; Hospital Nacional Niños, San Jose, San Jose, Costa Rica; Hospital Nacional de Ninos "Dr. Carlos Saenz Herrera", San Jose, San Jose, Costa Rica; St. Jude Children's Research Hospital, Germantown, TN

## Abstract

**Background:**

Early identification of the causative pathogen causing septic arthritis (SA) in children is crucial for guiding appropriate therapy; however, conventional culture methods may have low detection rates and delayed results. Molecular diagnostics have enhanced pathogen identification, potentially improving outcomes. This study aims to describe the clinical characteristics of children with SA following the implementation of the joint infection molecular panel in Costa Rica

**Table 1.**
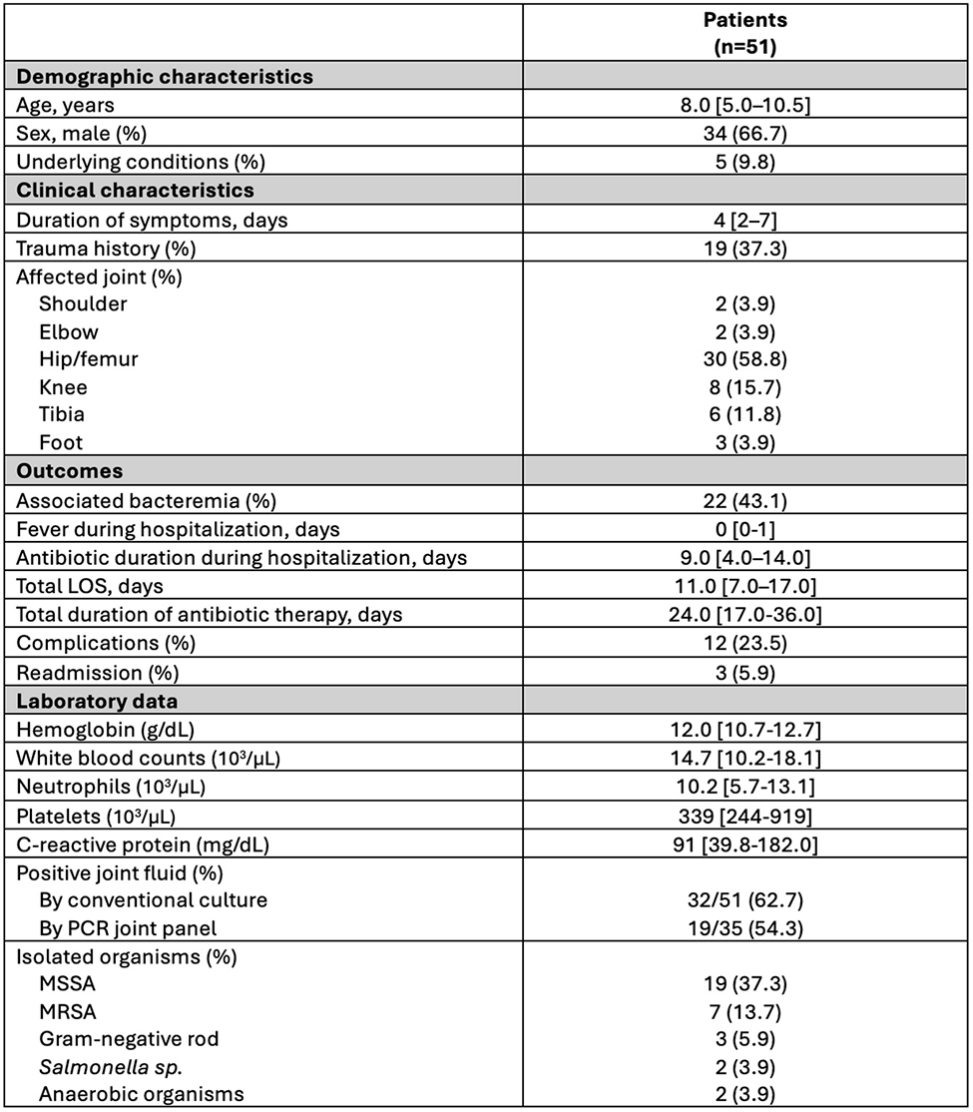
Clinical characteristics of patients admitted for septic arthritis Abbreviations: LOS, length of hospitalization; MSSA, Meticilin-sensible Staphylococcus aureus; MRSA, Meticilin-sensible Staphylococcus aureus. Data expressed as median [25-75% IQR] for continuous variables and as numbers (%) for categorical data

**Methods:**

In 2024-25, we conducted a retrospective observational study including children under 13 years of age hospitalized with SA at the only tertiary pediatric hospital. All patients underwent articular fluid aspiration for conventional culture and BIOFIRE® Joint Infection (JI) Panel testing. Clinical characteristics, treatment, and outcomes were documented

**Results:**

We enrolled 51 pediatric patients diagnosed with SA (median age: 8.0 [IQR: 5.0–10.5] years). Median duration of symptoms prior to admission was 4 days [2–7], and a history of trauma was reported in 19 (37%) cases. Lower extremity joints were predominantly affected, with the hip/femur (n=30, 59%), knee (n=8, 16%), and tibia (n=6, 12%) as most frequently involved. All patients underwent joint aspiration and received empirical intravenous antibiotics upon admission. The median length of hospitalization was 11 [7–17] days, with a median in-hospital antibiotic duration of 9 [4–14] days. Among 35 joint samples tested by both conventional culture and molecular diagnostics, there was no statistically significant difference in positivity rates (63% vs. 54%; p=0.63). *S. aureus* was the most common organism (54% isolated by culture vs. 49% by PCR, p=0.81), followed by gram-negative rod (6%), and *Salmonella sp.* (3%). The use of PCR panel provided results faster than conventional methods (2h vs. 48-72h). Overall, complications occurred in 12 (24%) patients (Table 1)

**Conclusion:**

While molecular diagnostics did not significantly improve pathogen detection rates compared to conventional cultures, they offered markedly faster turnaround times, which could support earlier pathogen-targeted therapy. Despite appropriate management, a proportion of patients experienced complications, highlighting the need for timely diagnosis and close follow-up

**Disclosures:**

All Authors: No reported disclosures

